# Circulation of prions within dust on a scrapie affected farm

**DOI:** 10.1186/s13567-015-0176-1

**Published:** 2015-04-16

**Authors:** Kevin C Gough, Claire A Baker, Hugh A Simmons, Steve A Hawkins, Ben C Maddison

**Affiliations:** School of Veterinary Medicine and Science, The University of Nottingham, Sutton Bonington, Loughborough, Leicestershire, LE12 5RD UK; ADAS UK, School of Veterinary Medicine and Science, The University of Nottingham, Sutton Bonington, Loughborough, Leicestershire, LE12 5RD UK; Animal and Plant Health Agency, Woodham Lane, New Haw, Addlestone, Surrey, KT15 3NB UK

## Abstract

Prion diseases are fatal neurological disorders that affect humans and animals. Scrapie of sheep/goats and Chronic Wasting Disease (CWD) of deer/elk are contagious prion diseases where environmental reservoirs have a direct link to the transmission of disease. Using protein misfolding cyclic amplification we demonstrate that scrapie PrP^Sc^ can be detected within circulating dusts that are present on a farm that is naturally contaminated with sheep scrapie. The presence of infectious scrapie within airborne dusts may represent a possible route of infection and illustrates the difficulties that may be associated with the effective decontamination of such scrapie affected premises.

## Introduction, methods and results

Transmissible Spongiform Encephalopathies (TSEs) are a group of fatal neurodegenerative diseases for which there is no effective treatment or cure. Examples of TSE infections affecting mammalian species include scrapie in sheep and goats, bovine spongiform encephalopathy (BSE) in cattle, chronic wasting disease (CWD) in deer and elk, and variant CJD (vCJD) in man. In each case the etiological agent is proposed to be a conformational isomer (PrP^Sc^) of the host encoded prion protein (PrP^C^) [1]. During a prolonged preclinical phase host PrP^C^ is converted into PrP^Sc^ which accumulates, particularly in the central nervous system, ultimately causing neuronal loss. The conversion of PrP^C^ to PrP^Sc^ confers several changes in the biochemical properties of the protein, such as a decreased solubility in detergents, and an increase in resistance to proteases and chemical denaturants.

For scrapie and CWD, prions are shed from infected animals via multiple routes and during preclinical and clinical stages of disease. For example, sheep infected with the scrapie prion secrete/excrete prions within faeces [2], saliva [3,4], urine [5] and skin [6]. Furthermore, parturient material is known to harbour high levels of scrapie infectivity [7,8]. The dissemination of this PrP^Sc^ coupled with its high stability leads to environmental reservoirs of infectivity. For example, it is known that premises that have housed scrapie-infected animals remain a potential source of infectivity for many years [9] and we have demonstrated that scrapie PrP^Sc^ can be detected on a range of surfaces within the farm providing likely sources of prion exposure [10].

Here, we use serial protein misfolding cyclic amplification (sPMCA) to investigate the presence of scrapie PrP^Sc^ within circulating dust material. sPMCA reproduces the prion replication cycle in vitro, to propagate prions with high sensitivity from small amounts of infectious material within a PrP^C^ “substrate” [11]. Samples were taken within an experimental farm with a high incidence of naturally transmitted scrapie [12]. Lambing was carried out within barns each spring where ewes and lambs were kept for up to a week before going to pasture. Samples were collected from within buildings and pasture that had not been used for holding animals for at least 12 months prior to sample collection. In addition, analogous samples were collected from a scrapie free environment (ARSU scrapie free sheep unit). This flock was maintained under a high level of biological containment to ensure that all animals were free from classical scrapie. Swab samples of surfaces were taken from areas within the scrapie affected barn that were inaccessible to sheep (horizontal and vertical surfaces at heights above 1.5 m) using a wetted foam swab (VWR). Briefly, swabs were moistened in sterile water and then an area of approximately 20 cm^2^ swabbed by passing the swab 10 times over the same area with each side of the swab. Four swabs were collected from each area sampled. In addition, samples of dust were taken that had collected in sterile petri-dishes over a 10-week period. For the scrapie affected farm, dust samples were taken from two barns that had previously housed scrapie affected animals, and from two barns that had never housed animals; one was used for storing equipment and the other for storing hay, these latter two barns were at least 50 m from the barns where animals had been housed. Dishes were contained within 10 mm mesh cages in order to prevent possible contamination by contact with birds and/or rodents, and collected dust at both floor level and a height of ~1 m. Similarly, to investigate the possibility that scrapie containing dust is circulated within pasture, two open ended polytunnels were set up at 30 m and 60 m from the open ends of the barns that had previously contained sheep. Within these, open petri dishes collected dust that settled within the pasture over this same 10-week duration.

Two swabs were used to sample all dust from a single petri dish. For swabs used to sample either petri dishes or directly sample surfaces on the farms, two swabs from the same sampling area or petri dish, were extracted into a phosphate buffer, and recovered using silicon dioxide as previously described [10]. When analysing the presence/absence of PrP^Sc^ in swabs of dust samples or from fomite surfaces, individual extracts (from 2 pooled swabs) were analysed. sPMCA was carried out within a 10% (w/v) sheep brain homogenate (VRQ/VRQ *Prnp* genotype at residues 136, 154 and 171) exactly as described previously [10]. Samples were subject to a total of 8 (surface swab samples) or 9 (dust samples) rounds of sPMCA. Amplification products were digested with 50 μg/mL proteinase K and resolved on a 12% SDS-PAGE gel and western blotted with detection of PrP^Sc^ by monoclonal antibody SHa31 as previously described [10]. Alternatively, dot blots were carried out by application of 2.5 μL digested amplified product that had been denatured in LDS, to a nitrocellulose membrane. Blocking, washing and immuno-labelling steps were carried out as for the western blot.

**Figure 1:**
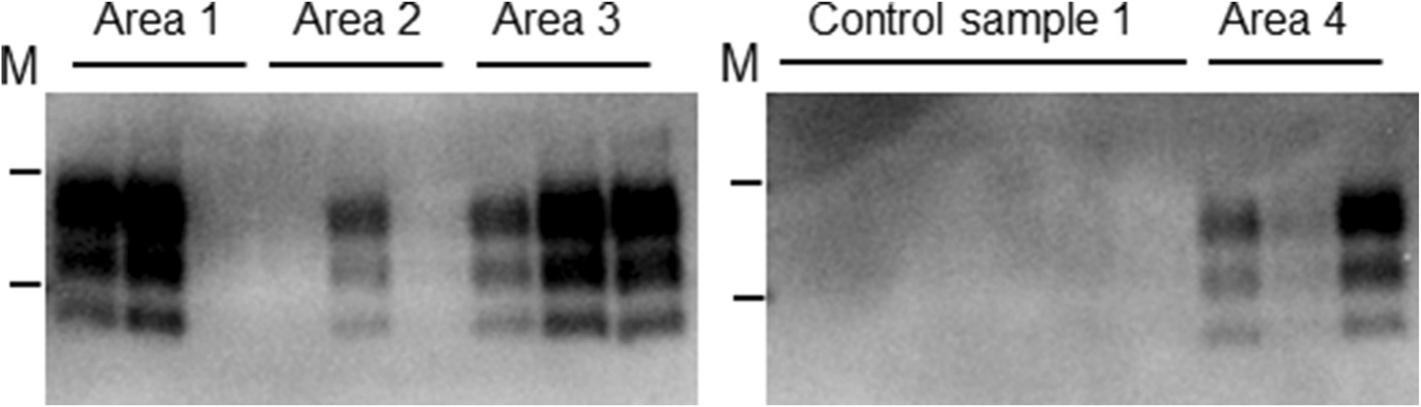
**sPMCA analysis of dust samples.** Example sPMCA western blot from dust samples taken from four surfaces (Area 1, 2, 3 and 4), inaccessible to direct contact with sheep, within a scrapie-affected barn were extracted and subject to sPMCA. Control sample 1: represent 6 individual reactions amplifying negative control extracts taken from a scrapie-free barn. PrP^Sc^ was detected using monoclonal antibody SHa31; M are 20 and 30 kDa molecular mass markers. In total 15/30 reactions from the scrapie affected farm were sPMCA positive, whilst 1/18 from the negative samples amplified, and presumed a false positive result.

**Figure 2:**
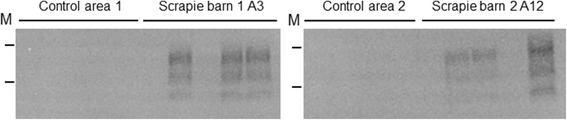
**Western blot of dust sPMCA analysis.** All sPMCA reactions at round 9 were analysed by dot blot (Table 1). Dot blot and western blot results were comparable, examples of 4 sample types subject to sPMCA are depicted as western blots. Control area 1 and control area 2 are samples collected from two areas of the scrapie negative control farm. Scrapie barn 1 area 3 and Scrapie barn 2 area 12 correspond to samples named in Table 1. PrP^Sc^ was detected using monoclonal antibody SHa31; M are 20 and 30 kDa molecular mass markers.

sPMCA analysis of swabs taken from surfaces that could not have been in direct contact from animals (horizontal and vertical surfaces above 1.5 m) demonstrated that sPMCA seeding activity could be detected (Figure 1). All areas sampled from the scrapie affected farm (6 extracts) seeded sPMCA amplification (total of 15/30 reactions) whereas for analogous samples collected from the scrapie free farm (3 extracts) only 1/18 reactions were positive. This observation suggested that dusts accumulating within buildings that have housed scrapie infected sheep (even when this was over 12 months previously) may contain scrapie prions. However, from these samples alone it cannot be ruled out that these surfaces were contaminated by direct deposition of saliva. In order to confirm that dust did harbour PrP^Sc^, dust collected on the sterile petri dishes from the scrapie affected farm were also analysed and were indeed found to seed sPMCA reactions even though the dust was collected over just 10 weeks (Figure 2, Table 1). Dusts collected from 5 different areas within 2 barns that had previously housed animals all supported sPMCA amplification (12 out of 25 reactions); in addition no scrapie PrP^Sc^ was detected within dust samples from two additional open barns that had no history of housing animals within this farm. For samples of dust within pasture, those that were collected 30 m from the scrapie positive buildings showed sPMCA positivity at a rate of 6/10 reactions, whereas there were no samples that were positive in those collected 60 m away from the buildings (0/10 reactions). Four analogous dust samples collected from barns that had housed sheep on a scrapie free farm did not support the amplification of scrapie PrP^Sc^ (0/20 amplifications). Overall the levels of sPMCA positivity from the dust samples collected within the scrapie affected farm are significantly different (*p* ≤ 0.005) from those reactions that used the control dust collected from the scrapie free farm (2-tailed Fishers exact test). A semi-quantitative assessment of sPMCA seeding activity within dust samples collected within petri dishes was also carried out. We determined the sPMCA rounds where both dilutions of scrapie brain homogenate and the positive dust samples were positive as described by Haley et al. [13]. Equivalent amounts of scrapie brain homogenate that were positive at the same level as the petri-dish dust samples equated to an upper limit of around 0.5 pg brain per reaction (data not presented). Taking into account the volume of the swab extract analysed and the total extracted area that was swabbed we estimate that over 70 days around 1 ng brain equivalent of scrapie seeding activity per m^2^ was deposited within the barn.

**Table 1:** **sPMCA analysis of collected dust samples**

**Dust collection area**	**Number positive reactions**
Control barn (10 dust sample extracts)	0/56
Equipment barn area 1 extract 1	0/5
area 1 extract 2	0/5
Hay barn area 1 extract 1	0/5
area 1 extract 2	0/5
Sheep barn 1 area 3	3/5
Sheep barn 1 area 4	1/5
Sheep barn 1 area 5	3/5
Sheep barn 2 area 11	2/5
Sheep barn 2 area 12	3/5
Polytunnel (30 m) extract 1	3/5
extract 2	3/5
Polytunnel (60 m) extract 1	0/5
extract 2	0/5

Dust samples collected in petri-dishes were extracted and then subject to sPMCA. Detailed are the individual samples that were extracted and subject to 9 rounds of sPMCA before PK digestion and analysis by dot blot. sPMCA products were scored positive/negative by dot blot after round 9.

## Discussion

We present biochemical data illustrating the airborne movement of scrapie containing material within a contaminated farm environment. We were able to detect scrapie PrP^Sc^ within extracts from dusts collected over a 70 day period, in the absence of any sheep activity. We were also able to detect scrapie PrP^Sc^ within dusts collected within pasture at 30 m but not at 60 m distance away from the scrapie contaminated buildings, suggesting that the chance of contamination of pasture by scrapie contaminated dusts decreases with distance from contaminated farm buildings. PrP^Sc^ amplification by sPMCA has been shown to correlate with infectivity and amplified products have been shown to be infectious [14,15]. These experiments illustrate the potential for low dose scrapie infectivity to be present within such samples. We estimate low ng levels of scrapie positive brain equivalent were deposited per m^2^ over 70 days, in a barn previously occupied by sheep affected with scrapie. This movement of dusts and the accumulation of low levels of scrapie infectivity within this environment may in part explain previous observations where despite stringent pen decontamination regimens healthy lambs still became scrapie infected after apparent exposure from their environment alone [16]. The presence of sPMCA seeding activity and by inference, infectious prions within dusts, and their potential for airborne dissemination is highly novel and may have implications for the spread of scrapie within infected premises. The low level circulation and accumulation of scrapie prion containing dust material within the farm environment will likely impede the efficient decontamination of such scrapie contaminated buildings unless all possible reservoirs of dust are removed. Scrapie containing dusts could possibly infect animals during feeding and drinking, and respiratory and conjunctival routes may also be involved. It has been demonstrated that scrapie can be efficiently transmitted via the nasal route in sheep [17], as is also the case for CWD in both murine models and in white tailed deer [18-20].

The sources of dust borne prions are unknown but it seems reasonable to assume that faecal, urine, skin, parturient material and saliva-derived prions may contribute to this mobile environmental reservoir of infectivity. This work highlights a possible transmission route for scrapie within the farm environment, and this is likely to be paralleled in CWD which shows strong similarities with scrapie in terms of prion dissemination and disease transmission. The data indicate that the presence of scrapie prions in dust is likely to make the control of these diseases a considerable challenge.

## Abbreviations

TSE: Transmissible spongiform encephalopathy
CWD: Chronic wasting disease
CJD: Creutzfeldt-Jakob disease
PrP^C^: Cellular prion protein
PrP^Sc^: Disease-associated prion protein
